# Future ocean conditions induce necrosis, microbial dysbiosis and nutrient cycling imbalance in the reef sponge *Stylissa flabelliformis*

**DOI:** 10.1038/s43705-023-00247-3

**Published:** 2023-06-14

**Authors:** Emmanuelle S. Botté, Holly Bennett, J. Pamela Engelberts, Torsten Thomas, James J. Bell, Nicole S. Webster, Heidi M. Luter

**Affiliations:** 1 Centre for Marine Science and Innovation, School of Biological, Earth and Environmental Sciences, University of New South Wales, Sydney, New South Wales Australia; 2 Australian Institute of Marine Science, Townsville, Queensland Australia; 3 Victoria University of Wellington, Wellington, New Zealand; 4 Cawthron Institute, Nelson, New Zealand; 5 Australian Centre for Ecogenomics, School of Chemistry and Molecular Biosciences, University of Queensland, Brisbane, Queensland Australia; 6 Australian Antarctic Division, Hobart, Tasmania Australia

**Keywords:** Environmental microbiology, Microbial ecology, Environmental sciences, Water microbiology, Molecular ecology

## Abstract

Oceans are rapidly warming and acidifying in the context of climate change, threatening sensitive marine biota including coral reef sponges. Ocean warming (OW) and ocean acidification (OA) can impact host health and associated microbiome, but few studies have investigated these effects, which are generally studied in isolation, on a specific component of the holobiont. Here we present a comprehensive view of the consequences of simultaneous OW and OA for the tropical sponge *Stylissa flabelliformis*. We found no interactive effect on the host health or microbiome. Furthermore, OA (pH 7.6 versus pH 8.0) had no impact, while OW (31.5 °C versus 28.5 °C) caused tissue necrosis, as well as dysbiosis and shifts in microbial functions in healthy tissue of necrotic sponges. Major taxonomic shifts included a complete loss of archaea, reduced proportions of Gammaproteobacteria and elevated relative abundances of Alphaproteobacteria. OW weakened sponge-microbe interactions, with a reduced capacity for nutrient exchange and phagocytosis evasion, indicating lower representations of stable symbionts. The potential for microbially-driven nitrogen and sulphur cycling was reduced, as was amino acid metabolism. Crucially, the dysbiosis annihilated the potential for ammonia detoxification, possibly leading to accumulation of toxic ammonia, nutrient imbalance, and host tissue necrosis. Putative defence against reactive oxygen species was greater at 31.5 °C, perhaps as microorganisms capable of resisting temperature-driven oxidative stress were favoured. We conclude that healthy symbiosis in *S. flabelliformis* is unlikely to be disrupted by future OA but will be deeply impacted by temperatures predicted for 2100 under a “business-as-usual” carbon emission scenario.

## Introduction

Marine sponges (phylum Porifera) are highly diverse, filter-feeding benthic animals, which play essential roles in the ocean, such as habitat formation, benthic-pelagic nutrient coupling and secondary metabolite production [1–3]. Sponges are also a source of remarkable microbial diversity, together forming what is referred to as “sponge holobionts” [4, 5]. Far from being passive components, the bacterial and archaeal partners play key roles that contribute to host health and survival [4, 6] including energy provision to the host, waste removal, amino acid and secondary metabolite production and chemical defence [2, 7–10].

Over the last decade, temporally and geographically stable sponge-microbe partnerships have been uncovered in most species analysed [11–13]. However, while microbial communities associated with sponges are highly stable under optimal environmental conditions, they can shift when the host is exposed to environmental stressors. Globally, oceans have absorbed nearly a third of the excess CO_2_ released into the atmosphere through anthropogenic activities, compared to pre-industrial times [14]. This has already resulted in OW of 0.88 °C and OA from pH 8.2 to pH 8.1 [14]. Disruption of the taxonomic composition of sponge microbiomes has been reported in several species in response to both thermal stress [15–18] and OA [19–21]. Whilst fewer studies have investigated the consequences of OW and OA on the microbial functional repertoire, perturbation has been found in at least two tropical species. For instance, the microbiome of *Rhopaloeides odorabile* exhibits reduced expression of transporters involved in the uptake of sugars and peptides when the sponge is exposed to elevated temperature [22] and the microbiome of *Coelocarteria singaporensis* living under OA conditions at natural CO_2_ seeps has a higher potential for inorganic carbon fixation and nitrogen cycling, compared to unimpacted reefs [21].

Historically, studies exploring the effects of climate change on sponge holobionts have focused on exposure to either OW or OA [23]. More recently, simultaneous OW and OA exposure have been applied to investigate the host’s physiological response (reviewed in ref. [24]), or the microbiome composition [25, 26]. To our knowledge, only one study has recently explored the simultaneous impacts of OW and OA on both the sponge and its associated microbiome, focusing on the immune response [27]. Given that sponges will continue to face both stressors in the coming decades, and that microorganisms play key roles in sponge health, it is urgent to determine the effects of concomitant OW and OA on these partnerships [28].

The tropical sponge *Stylissa flabelliformis* constitutes an ideal model species to examine these aspects of sponge symbiosis. Populations of *S. flabelliformis* are sensitive to life-long exposure to pH levels predicted to become global averages by 2100, and which are already measured at natural CO_2_ seeps in Papua New Guinea [21], and are also susceptible to thermal stress [29]. Additionally, the microbiome associated with *S. flabelliformis* is well characterized [13, 30], and specific microorganisms have been identified as symbionts of *S. flabelliformis* [31], which provides a solid basis for data interpretation.

Here we present a comprehensive view of the sponge holobiont’s response to OW and OA predicted to occur within the next eighty years. We assessed sponge health and used a combination of metagenomics and targeted genomics to reveal the potential impact of both stressors on sponge symbioses. Specifically, we determined (i) to what extent changes in sponge health upon exposure to both stressors are reflected in the microbial composition of *S. flabelliformis*, (ii) how OW and OA affect the overall microbial functional repertoire, (iii) if specific functional signatures originating from the microbial communities could explain potential effects on the sponge holobiont.

## Methods

### Experimental procedure

Specimens of the common Great Barrier Reef sponge species *Stylissa flabelliformis* were collected from Davies Reef (18°82’S, 147°65’E) and transported to the National Sea Simulator (SeaSim) at the Australian Institute of Marine Science (AIMS) as part of a study investigating sponge health and physiology in response to environmental stress, and describing the methods in detail [29, 32]. Briefly, specimens were cut into smaller samples and maintained in flow-through aquaria to recover for four weeks, followed by 3 days at ambient experimental conditions at 27 °C and pH 8.0. Subsequent experimental temperature and pH were respectively achieved by mixing 22 °C and 36 °C seawater and by supplying seawater with CO_2_ gas; accurate conditions were maintained using a fully automated system within the SeaSim, while ensuring 100% water replacement every hour, as described in detail in refs. [29, 32]. Gradual changes in seawater temperature and pH were applied following the schedule detailed in Table S1, to represent present day conditions (28.5 °C, pH 8.0) or ocean conditions predicted for 2100 under RCP8.5 (31.5 °C, pH 7.6, ref. [33]). A fully factorial design was applied, and sponges were therefore exposed for eight weeks to 28.5 °C or 31.5 °C in combination with pH 8.0 or pH 7.6 (i.e., four treatments, see Table S2).

### Sampling and sponge health assessment

Percentage tissue necrosis was assessed after eight weeks of exposure, using Image J as described previously [29]. Tissue biopsies were then immediately collected from three samples for each of the four treatment combinations (*n* = 12 total), using sterile gloves and scalpel blades. Healthy portions of tissue were selected when sampling necrotic sponges. Samples were snap frozen in liquid nitrogen and stored at −80 °C until further processing for the analysis of microbial communities.

### Microbial cell enrichment, DNA extraction and sequencing

To separate microbial cells from eukaryotic cells, each sample weighing ~1.5 g (wet weight) was placed into calcium and magnesium free seawater (CMFSW composed of 0.4 mM NaCl, 10 mM KCl, 7 mM Na_2_SO4 and 0.5 mM NaHCO_3_), thoroughly homogenised using a SilentCrusher M (Heidolph, Schwabach, Germany) and subjected to a series of centrifugation and filtration steps following the procedures detailed in ref. [21]. DNA was subsequently extracted from the microbial cells using the UltraClean Microbial DNA Isolation Kit (MoBio Laboratories, Inc., Carlsbad, USA), following the manufacturer’s protocol. Quality and quantity of DNA were assessed using gel electrophoresis (1% agarose gel containing ethidium bromide), spectrophotometry (NanoDrop 2000, ThermoFisher Scientific, Waltham, USA) and fluorimetry (Qubit, ThermoFisher Scientific, Waltham, USA). DNA was subjected to a Nextera XT library preparation and downstream paired-end (2 × 300 bp) sequencing on the Illumina MiSeq platform (Ramaciotti Centre for Genomics, Sydney, Australia).

### Bioinformatics

GraftM (https://github.com/geronimp/graftM_gpkgs) was used to taxonomically classify the reads. GraftM employs gene-specific packages to identify taxa using hidden Markov models (HMMs) or DIAMOND databases to classify these sequences by placing them into pre-constructed gene trees [34].

For functional annotation, raw sequences were quality trimmed using Prinseq [35] and assembled using IBDA-UD v1.1.1 [36] with default settings and the pre-correction option (Table S3). Reads were mapped to assembled contigs (>200 bp) using Bowtie 2 [37] and SAMtools [38] to create coverage files. Fasta and coverage files were subsequently uploaded to IMG/M [39] for annotation using standard gene calling methods from IMG [40]. Estimated gene copy number of KEGG Orthology terms or KOs [41] was exported for each sample and used in downstream analyses. All contigs identified as being “eukaryotic” by IMG were discarded before further analyses. Pfam of interest (see results section) were also retrieved. Finally, the taxonomic origin of KOs was retrieved via IMG for functions of interest.

### Statistical analysis

Data wrangling and all figures were made using R [42] and packages tidyverse and ggplot2 [43, 44]. Analysis of variance (ANOVA) was used to test the effect of pH, temperature and the interaction between the two factors on host health using the R aov function. For the microbiome composition, since a relatively low number of taxa was identified (see below), they were all retained to assess the impact of OW, OA and the combination of factors. Counts were converted into percentage relative abundances (RA) and square-root transformed to produce a Bray-Curtis dissimilarity matrix, which was analysed with Permutational analysis of variance (PERMANOVA) with 9999 permutations on PRIMER7/PERMANOVA + (Plymouth, UK). Given the PERMANOVA results showing no effect of pH, raw counts were then used to determine specific changes in the composition as a result of temperature treatment only. Due to the large number of structural zeros in the dataset, we used the ANCOM-BC R package to identify taxa that varied with temperature treatments [45]. Default parameters were used to run the analysis with the “Holm” adjusting method.

For the functional analysis, KOs found in only one sample were removed, resulting in a total of 5370 KOs to be analysed as a multivariate dataset. PERMANOVA was conducted with 9999 permutations on Bray-Curtis distances of standardized and square-root transformed data to assess the effects of OW, OA and potential interactions on the functional profiles of *S. flabelliformis*. Based on the PERMANOVA results, the EdgeR package [46] was used to determine which KO significantly differed between the 28.5 °C and 31.5 °C conditions. Default settings were used except for “large.n”, which was set at 5. The EdgeR default filtration step, which ensures that only KOs with sufficient counts are tested, resulted in a total of 1863 KOs being kept for statistical testing, which exhibited a minimum count number of 76 across all samples. The 913 significantly different (false discovery rate of <0.05) KOs (KO_sig_) that fell into higher level categories other than “Brite hierarchies” and “Unknown” or “Undefined” were used to generate the plot displayed on the figure (see Supplementary results for further detail).

To further identify the biological processes impacted by OW, KEGG modules were identified within the dataset using the “annotate” and “classify” functions of enrichM (https://github.com/geronimp/enrichM). Only modules that were at least 70% complete (abbreviated mod_70_) were kept for further analysis. KOs identified across all metagenomes fell into 219 prokaryotic mod_70_. A total of 192 mod_70_ were kept for statistical analyses by EdgeR, which was run with the same settings as for the KOs analysis.

Additional EdgeR statistical analysis was conducted on Pfam annotations [47] to specifically investigate potential changes in the RA of eukaryotic-like proteins (ELPs), which are suspected to play a role in maintaining sponge symbionts within the holobiont, by allowing phagocytosis evasion [48, 49]. We present results pertaining to transporters together with ELPs as both functions are widely considered markers of sponge symbiosis by facilitating chemical and molecular interactions between host and symbionts [28].

### Genome-based analysis

Two sets of results required further investigation: *(*i) the distribution of the ELP tetratricopeptide repeats (TPRs) among microorganisms associated with *S. flabelliformis*, and (ii) the presence in these microorganisms of genes involved in rhamnose biosynthesis, a molecular marker usually depleted in symbiotic microorganisms [28]. To conduct this finer-scale analysis, we assessed the presence of these functions in 28 published Metagenome Assembled Genomes (MAGs) associated with *S. flabelliformis* [8].

## Results

### Microbial communities mirror sponge health response with sensitivity to OW but not OA

**Fig. 1:**
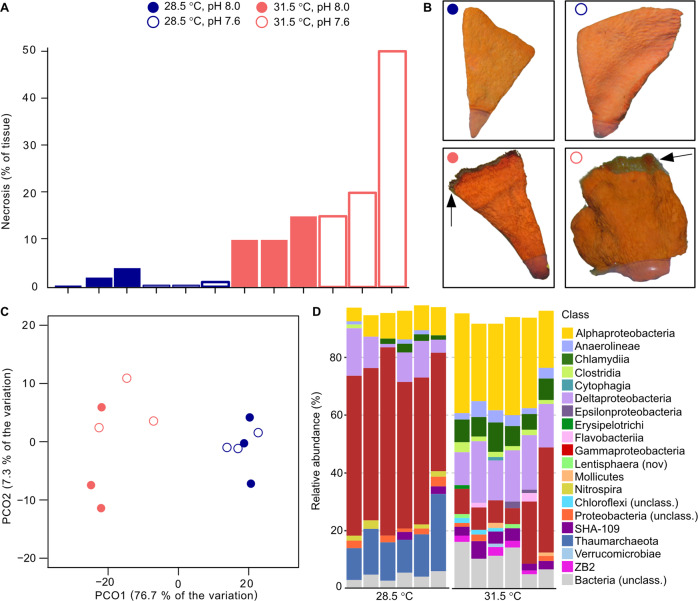
Holobiont’s health and overview of the microbiome composition. **A** Percentage of necrosed tissue on samples at the end of the 8-weeks experiment. **B** Pictures of representative samples for each of the four combined treatments; arrows point to necrotic tissue. **C** PCoA plot of microbiome structure using GraftM taxonomic classification at the class level. **D** Taxonomic classification of the microbiome. Data shown for classes >1%.

Exposure to 31.5 °C for eight weeks had a significant effect on sponge health when compared to 28.5 °C as reported previously [29]. Samples exposed to OW showed significantly higher percentage of tissue necrosis (ANOVA, F_1,8_ = 11.473, *p* = 0.009), whereas no visible effect was evident at low pH (ANOVA, F_1,8_ = 1.819, *p* = 0.214) and there was no interactive effect of OW and OA (ANOVA, F_1,8_ = 2.718, *p* = 0.138) (Fig. 1A, B).

The taxonomic structure of the microbiome of *S. flabelliformis* significantly shifted in response to elevated temperature (PERMANOVA, Pseudo-F_1,8_ = 16.661, *p* = 0.0017, Fig. 1C), but there was no significant effect of pH and no interaction between the two factors. The higher temperature treatment induced statistically significant changes in the RA of abundant taxa (Fig. 1D, Table S4 and Supplementary data spreadsheet “SOM_taxonomy”). Specifically, the phylum Thaumarchaeota (now Thermoproteota, [50]) was only detected at 28.5 °C where it represented more than 10% of the community, and representatives of the order Thiohalorhabdales (Gammaproteobacteria) decreased from 21.3% to only 2.2% at higher temperature. Notably, Nitrospira were present in five of the six samples at 28.5 °C (representing 1.5% of the community on average) but were completely absent at 31.5 °C. Conversely, the proportion of several Alphaproteobacteria taxa increased at higher temperature with the order Rickettsiales changing from 7.8% to 21.3%, while the family Rhodobacteraceae increased by more than 30-fold, from 0.1 to 3.3%. Finally, the order Chlamydiales represented 1% of the community at 28.5 °C and 4.9% under OW. Given the dramatic loss of Thaumarchaeota at high temperature, we compared the taxonomy of these organisms to that of Thaumarchaeota from 81 sponge species [13] and found that the archaeal phyla present in *S. flabelliformis* at 28.5 °C were those most commonly occurring in marine sponges (see Supplementary results for further detail).

### Major shift in microbial functional potential at high temperature

**Fig. 2:**
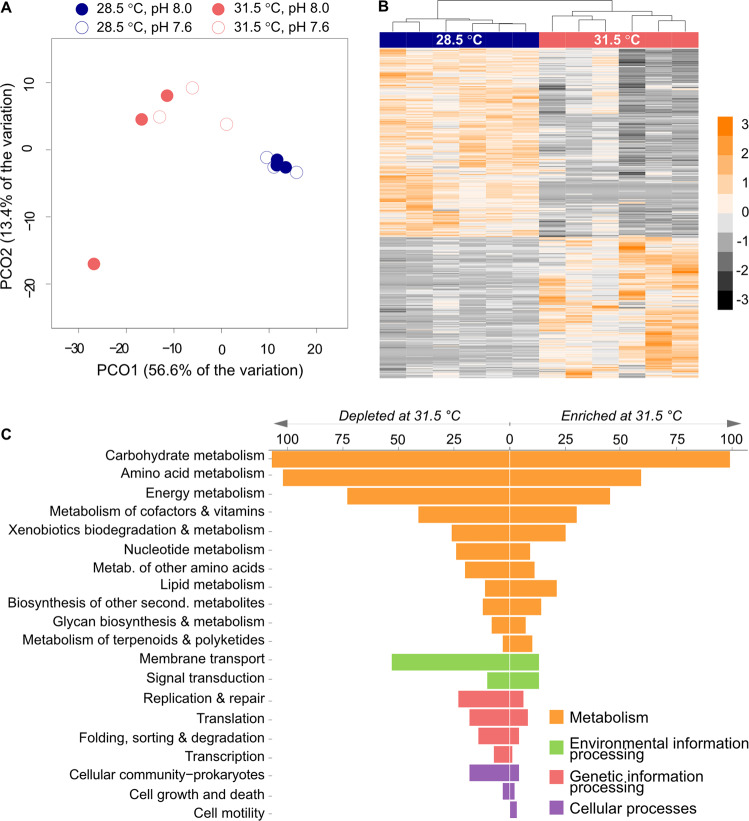
Overview of the functional potential of microorganisms associated with *S. flabelliformis* according to temperature and pH treatments. **A** Sample partitioning visualised as a PCoA on the KEGG Orthology terms (KOs). **B** Heatmap on the relative abundance of the 913 KOs with significantly different abundances according to temperature. Data were scaled by row and colour scale represents standard deviation. **C** Classification of the significantly different KOs (KO_sig_) into KEGG pathway categories of higher levels. Values indicate the number of KOs assigned to each category.

Consistent with the taxonomic shifts, the overall functional potential of the microbiomes changed with temperature (PERMANOVA, pseudo-F_1,8_ = 9.3464, *p* = 0.0028) but not pH (PERMANOVA, pseudo-F_1,8_ = 1.2967, *p* = 0.2758), and no interaction was identified (PERMANOVA, pseudo-F_1,8_ = 1.404, *p* = 0.2282) (Fig. 2A). A total of 913 KOs, representing close to half (49%) of the KOs tested, exhibited significantly different RA between 28.5 °C and 31.5 °C (Supplementary data spreadsheet “SOM_KOs”). Within these significantly different KOs (KO_sig_), 521 (57%) were under-represented at 31.5 °C and 392 (43%) over-represented (Fig. 2B). Classification according to KEGG pathways (see Supplementary notes) revealed that the vast majority (79%) of KO_sig_ belonged to KEGG metabolism pathways and 58% were associated with energy metabolism and the metabolism of carbohydrates, amino acids, cofactors and vitamins (Fig. 2C). Notably, 9.3% of KO_sig_ were assigned to signal transduction and membrane transport.

Analysis of KEGG modules representing entire pathways encompassing several genes, revealed that 79 mod_70_ (modules with at least 70% completeness) were enriched in one of the temperature treatments, representing 41.1% of the modules statistically tested (Table S5). Confirming the pattern found at the KO level, half (50.6%) of these modules fell into categories related to metabolism (including 41.8% involved in energy metabolism and the metabolism of amino acids, carbohydrates, cofactors and vitamins), whilst 39.2% were related to environmental information and processing, and 5.1% to genetic information and processing (Supplementary data spreadsheet “SOM_modules” for the complete list of modules).

### Temperature effects on markers of symbiosis

**Fig. 3:**
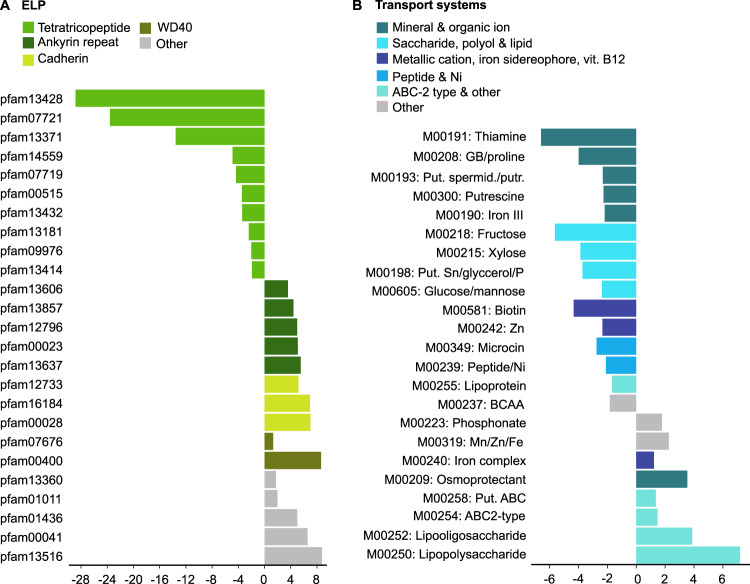
Effect of temperature on markers of symbiosis. (**A**) Eukaryotic-like proteins (ELPs). (**B**) Transport systems. X-axis represents fold-change. Negative numbers represent depletion at 31.5 °C, while positive numbers represent enrichment at 31.5 °C. Abbreviations are “GB” for glycine betaine, “put.” for putative, “spermid.” for spermidine, “putr.” for putrescine, “BCAA” for Branch-chain amino acids.

Temperature increase significantly affected three types of symbiosis markers considered to play key roles in maintaining the partnership between the host and its associated microbes. First, 60% of KOs representing Eukaryotic-Like Proteins (ELPs, retrieved from the Pfam database) were enriched at 31.5 °C, and a striking dichotomy was found depending on which type of ELP was examined: all tetratricopeptide domains (TPR) were less abundant at high temperature with fold-changes ranging from 2 to 29, whereas all others (including ankyrin repeats, cadherin-like, leucine-rich domains, pyrrolo-quinoline quinone—or PQQ—domains and WD40 domains) showed the opposite trend (Fig. 3A). Further analysis revealed that although 14% of the 28 published MAGs from *S. flabelliformis* were archaeal, these bins carried 45% of the Pfams terms related to TPRs, indicating that archaea associated with *S. flabelliformis* are enriched in TPRs compared to other microbes associated with this sponge species (Supplementary data spreadsheet SOM_genome_analysis, tab#1).

Second, 65.2% of the 23 significant mod_70_ representing transport systems were enriched at 28.5 °C (Fig. 3B). Every module that was more abundant at control temperature exhibited a fold-change in RA of at least 1.5, which was the case for only three of the eight modules enriched under OW. All modules related to carbohydrate transport systems (glucose, mannose, fructose and xylose) as well as peptide (microcin) and nickel transport were at least twice as abundant at control temperature as they were under OW. The same result was found with five of the six modules representing the transport of thiamine (M00191), the osmoprotectants glycine betaine and proline (M00208), the nitrogenous compounds spermidine and putrescine (M00193 and M00300) as well as iron III (M00190).

Third, the module representing rhamnose biosynthesis (M00793) was 2.4-times more abundant at 31.5 °C (Supplementary data spreadsheet “SOM_modules”). No shift in the taxonomy of the KOs representing rhamnose biosynthesis was evident in our dataset, with Alphaproteobacteria as the dominant taxon carrying these genes at both temperatures (Table S6). We hypothesised that this taxonomic stability resulted from two factors leading to an over-representation of M00793 at 31.5 °C: (i) archaea associated to *S. flabelliformis* generally lack the capacity to synthesise rhamnose and (ii) the RA of bacteria increased compared to that of archaea at 31.5 °C. To test this hypothesis, we examined 28 MAGs from *S. flabelliformis* (from ref. [8]) and found that, while 25% of bacterial MAGs encoded the complete module for rhamnose biosynthesis, none of the four archaeal MAGs did (Supplementary data spreadsheet SOM_genome_analysis, tab#2).

### Energy metabolism and biosynthesis of amino acids, cofactors and secondary metabolites

**Fig. 4:**
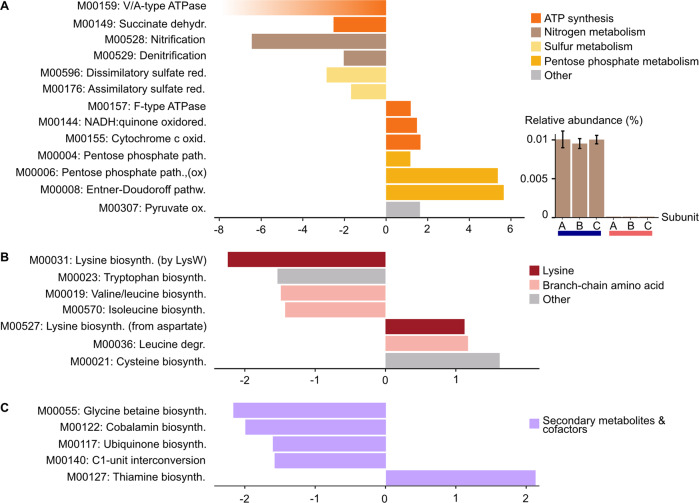
Elevated temperature induces loss of ammonia oxidation and affects modules pertaining to metabolism. **A** (left panel) Energy metabolism modules. **A** (right panel) Relative abundance of KOs representing ammonia monooxygenase subunits at 28.5 °C (blue) and 31.5 °C (red). **B** Amino acids modules. **C** Secondary metabolites modules. All panels except A-right show fold-change on the x-axis, with negative numbers representing depletion at 31.5 °C and positive numbers representing enrichment at 31.5 °C. The colour gradient added to M00159 represents the absence of this module at 31.5 °C.

Many aspects of energy and nutrient metabolism were impacted by elevated temperature with 13 modules involved in oxidative phosphorylation, nitrogen and sulphur metabolism showing enrichment between treatments (Fig. 4). Within these, five modules representing reactions within the electron transport chain were significantly affected by temperature. The largest change was found with a suite of genes encoding V/ATPases for ATP synthesis in archaea (M00159). This module was 100% complete at 28.5 °C, whereas only three out of the nine KOs were found at 31.5 °C, and in only one sample. All genes were carried by Thaumarchaeota at 28.5 °C, explaining the absence of this module under thermal stress (Table S7). The lack of Thaumarchaeota also explained the reduced functional potential at 31.5 °C for the conversion of succinate to fumarate, catalysed by succinate dehydrogenases (M00149, EC 1.3.5.1) as part of the oxidative phosphorylation process or the TCA cycle (2.5-fold decrease in RA), with archaea comprising an average of 22% of the genes’ taxonomy at 28.5 °C, but always 0% at 31.5 °C (Table S7). In addition, modules representing cytochrome c oxidases (M00155) and NADH:quinone oxidoreductases (M00144) as well as F-type ATPases were enriched at 31.5 °C (fold-changes of 1.7, 1.5 and 1.2, respectively).

The potential for nitrogen and sulphur metabolism decreased at 31.5 °C. Two modules representing nitrification and denitrification were reduced under OW by a factor of six and two, respectively (Fig. 4A, left panel). Genes encoding ammonia monooxygenase subunits, which catalyses the first step of nitrification (ammonia oxidation to nitrite), were only found at 28.5 °C (Fig. 4A, right panel) and carried exclusively by Thaumarchaeota (Table S7). The lower potential for denitrification at 31.5 °C was also partially due to the loss of Thaumarchaeota carrying nitrite reductase NO-forming nirK (EC:1.7.2.1). Nitrospira were major contributors to the trend as they carried the gene encoding nitrate reductase/nitrite oxidoreductase at 28.5 but not at 31.5 °C (Table S7). Two modules related to dissimilatory and assimilatory sulphate reduction exhibited a 2.9-fold and 1.6-fold decrease at 31.5 °C, respectively. A reduced proportion of Betaproteobacteria appeared responsible for the lower potential for the dissimilatory pathway via adenylylsulfate reductase (EC:1.8.99.2) (Table S7), while the taxonomic origin of genes encoding the assimilatory pathway was inconclusive.

The majority of the nine significantly different modules representing carbohydrate metabolism were enriched at 31.5 °C (Fig. 4B). This was particularly striking with modules representing the complete pentose phosphate pathway (PPP, M00004), the oxidative branch of the PPP (oxPPP, M00006) and the Entner-Doudoroff pathway (EDP, M00008), with fold changes of 1.2, 5.4 and 5.7, respectively. This resulted from the gene encoding glucose/mannose-6-phosphate isomerase (EC 5.3.1.9, as part of the non-oxPPP) being exclusively carried by Thaumarchaeota and only found at 28.5 °C, while ribose 5-phosphate isomerase (EC 5.3.1.6, also part of the non-oxPPP) was only present at 31.5 °C and exclusively carried by Alphaproteobacteria (Table S7). Whilst the PPP is ubiquitous, oxPPP and EDP have so far not been found in archaea [51, 52], explaining the different fold-changes between modules representing PPP on one hand (1.2-fold), and oxPPP and EDP on the other (5.4- and 5.7-fold).

High temperature generally reduced the potential for amino acid (AA) biosynthesis (Fig. 4B), with five modules representing the biosynthesis of (i) branch-chain amino acids (BCAA) valine, leucine and isoleucine (M00570 and M00019 with fold-changes of 1.4 and 1.5 respectively) (ii) tryptophane (M00023, with a fold-change of 1.5), and (iii) lysine (M00031 with a fold change of 2.2). In addition, one of the only three modules enriched at 31.5 °C represented the degradation of leucine (M00036, fold-change of 1.2), while a second exhibited a low fold-change (M00527, fold-change of 1.1). These results largely reflect the taxonomic shift observed under OW. For instance, M00031 represents a pathway involving the archaeal gene LysW, previously found in the sponge *Coelocarteria singaporensis* [21] and these genes were carried exclusively by Thaumarchaeota (Table S7). Similarly, the shared section of the two modules representing BCAA biosynthesis (enzymes EC 2.2.1.6, 1.1.1.86, 4.2.1.9 and 2.6.1.42) was encoded by Thaumarchaeota, Nitrospira, Rhodobacteraceae and Chromatiales at 28.5 °C, whilst no read was assigned to the Thaumarchaeota or Nitrospira at 31.5 °C (Table S7). Finally, within the pathway converting chorismate into tryptophane, KOs representing four of the five required enzymes were carried by Thaumarchaeota (11–40%) at 28.5 °C, but only by bacterial taxa at 31.5 °C (Table S7).

Modules involved in the metabolism of several cofactors and vitamins were depleted under high temperature, including biosynthesis of glycine betaine (M00055, fold-change of 2.2), cobalamin (vitamin B12, M00122, fold-change of 2) and ubiquinone (M00117, fold-change of 1.6), as well as the interconversion of the one-carbon compound folate (vitamin B9, M00140, fold-change of 1.6) (Fig. 4C). Only one set of genes encoding the thiamine biosynthesis pathway (vitamin B1, M00127), was 2.1-times more abundant under OW, likely the result of the increase in Alphaproteobacteria at that temperature (Table S7). The reduced potential for the production of glycine betaine (GB) was caused by lower proportions of Gammaproteobacteria, with half of the genes carried by Gammaproteobacteria at 28.5 °C, but by Alphaproteobacteria at 31.5 °C. Reduced proportions of Thaumarchaeota at 31.5 °C were responsible for the lower RA of several genes encoding cobalamin and ubiquinone biosynthesis pathways, and for two of the three genes involved in the capacity to use folate for the transport of one-carbon (C1-) groups (Table S7).

Additional results on carbohydrate metabolism are reported in the Supplementary notes.

## Discussion

### Increased temperature induces dysbiosis in S. flabelliformis

*S. flabelliformis* was adversely affected by conditions of OW projected for 2100 but unaffected by OA. The response of the microbiome to OW mirrored that of the host with dysbiosis involving a complete loss of Thaumarchaeota, a dramatic decline in the proportion of Thiohalorhabdales and a concomitant enrichment in Alphaproteobacteria. Conversely, the stability of the microbiome under OA corroborates previous observations of natural populations of *S. flabelliformis* living in acidified waters in shallow volcanic CO_2_ seeps in Papua New Guinea [20]. The microbial composition of healthy *S. flabelliformis*, dominated by Thaumarchaeota and Gammaproteobacteria (and to a lesser extent Deltaproteobacteria), is highly consistent with that of individuals collected thousands of kilometres away in Papua New Guinea and Indonesia [21, 53], as well as previous research confirming archaea as obligate symbionts [31, 54]. The 31.5 °C exposure therefore impacted consistently abundant and important taxa in *S. flabelliformis*.

Whilst elevated seawater temperature has previously been shown to impact the composition and structure of the sponge microbiome, most studies have investigated the effects of acute stress (i.e. rapid and extreme changes in environmental conditions) and holobiont responses are therefore hard to interpret within an ecological context. For example, shifts in microbial communities associated with *Rhopaloeides odorabile* occurred after exposure to a minimum of +5 °C in adults resulting in necrotic individuals after four days [17, 22] and +6 °C in larvae [55]. A milder climate change regime (+4 °C and −0.4 pH units from 8.0 to 7.6) was employed in a study conducted on *Neopetrosia compacta* and *Leucetta chagosensis* [27] but despite sponges exhibiting necrosis after two days, experimental conditions had no effect on the microbial community, highlighting the species-specific response of sponge microbiomes to OW and OA conditions.

Of particular interest is a study conducted on *Scopalina sp*., which was exposed to increases in temperature (+7 °C over 20 days) and developed disease symptoms over time, with preceding changes in the taxonomic structure of the microbiome [15]. These findings are consistent with ours, whereby restructuring of the microbial communities occurred in healthy-looking tissue of temperature-stressed individuals. This pattern suggests that temperature-induced microbial dysbiosis is one of the driving factors of declining host health and highlights the utility of the *S. flabelliformis* microbiome as a sensitive indicator of host health.

Our report of dysbiosis in a reef sponge in response to a 3 °C increase in seawater temperature is timely. Climate change is expected to impact sponge populations, with widespread sponge mortalities due to abnormally high seawater temperatures occurring in Indonesia, the USA and across the Mediterranean [56–59]. In addition, marine heatwaves of this magnitude have already been observed in Australia, with temperatures 1.5–3 °C above normal, along the East coast between November 2015 and February 2016 [60]. With such extreme events predicted to occur more frequently in the coming decades [14], natural populations of *S. flabelliformis* face a challenging future.

### Thermal stress weakens host-microbiome interactions at the molecular level

Temperature-induced dysbiosis in *S. flabelliformis* involved changes in the RA of nearly half of the genes analysed, demonstrating a major impact of OW on the functional repertoire of the sponge microbiome. Enrichment profiles for three types of symbiosis markers (ELPs, transporters and rhamnose biosynthesis) were particularly strong.

Over the last decade, ELPs have been hypothesised to allow microorganisms to evade predation from sponge cells by interfering with phagocytosis via protein-protein interactions. More specifically, symbionts of *Cymbastella concentrica*, *Scopalina sp*. and *Tedania anhelens* expressed ELPs cadherin, tetratricopeptides and ankyrin repeats [61]. In addition, ELP classes ankyrin repeats, tetratricopeptides, as well as NCL-1, HT2A and Lin-41 sequence repeats (NHL) from the sponge *Cymbastella concentrica* were shown to reduce phagocytosis when expressed in *E. coli* exposed to amoeba [48, 62]. Finally, ankyrin repeats encoded by sponge-associated phages were found to reduce phagocytosis of bacteria [49]. Certain types of ELPs are enriched in specific lineages while others are ubiquitous [8]. Here we identified the depletion of TPRs at 31.5 °C resulting from the lack of archaea at this temperature, and the concomitant enrichment of all other ELPs, indicating changes in the nature of host-symbiont interactions.

Genes encoding transporters are also often enriched in the genomes of sponge-associated microbes compared to free-living ones [63, 64]. This feature may have been selected for over time to sustain the complex nutrient exchanges between the host and its microbiome. The lower functional capacity for metabolic exchange via transport systems at 31.5 °C compared to 28.5 °C therefore suggests that the overall proportion of symbiotic microbes decreased under OW.

A weakening in the molecular interactions between the host and associated microorganisms under temperature stress was further evidenced by the enhanced potential for rhamnose biosynthesis. A lack of genes encoding rhamnose biosynthesis pathways is increasingly considered a distinguishing feature of sponge-associated symbionts [28]. This hypothesis was first proposed based on the observation that *Ca*. Synechococcus spongiarum from various sponge species lacked the genetic capacity to synthesise L-rhamnose, which in free-living *Synechococcus* is a key component of the O-antigen of lipopolysaccharides, but also an important antigen recognised by the immune system to trigger phagocytosis [65, 66]. A lack of rhamnose biosynthesis might therefore represent an evolutionary trait that allows sponge-associated microbes to evade phagocytosis [54, 65, 67]. In addition, all genomes from the thaumarchaeal symbionts of *Petrosia ficiformis* and *Hymedesmia (Stylopus) methanophila* largely lack COGs associated with rhamnose biosynthesis compared to free-living Thaumarchaeota [54]. The absence of rhamnose biosynthesis modules in the Thaumarchaeota MAGs from *S. flabelliformis*, combined with an enrichment of genes encoding this pathway at 31.5 °C, further confirm that sponges harboured a lower proportion of truly symbiotic taxa at high temperature.

Loss of symbionts and their associated functions have also been reported in necrotic *Rhopaloeides odorabile* following thermal stress [22], including a reduced potential to take up exogenous compounds and a shift in ELPs (namely fibronectin III domains and collagen binding domains), consistent with our findings for *S. flabelliformis*. Additional work is now required to understand the ubiquity of breakdown in these functional traits upon exposure to elevated temperature across the wide diversity of sponge species.

### Dysbiosis disrupts nutrient cycling

Dysbiosis severely impacted the metabolic capability of the microbiome, in particular via the complete loss of Thaumarchaeota. Sponge-associated Thaumarchaeota can conduct energy-efficient carbon fixation with potential organic carbon transfer to the host, conversion of urea into ammonia, ammonia oxidation into nitrite and the production of cobalamin (vitamin B12) [21, 31, 54, 68, 69]. Here the absence of this taxon resulted in a loss of ammonia oxidation capacity, corroborating previous findings that Thaumarchaeota are the only ammonia-oxidising microorganisms in *S. flabelliformis* [7, 8].

A loss of Nitrospira at elevated temperature was responsible for the overall declining RA of the denitrification module, via a lower proportion of genes encoding nitrate reductase/nitrite oxidoreductase, but whilst this enzyme can catalyse nitrate reduction, Nitrospira primarily use it to perform the second step of nitrification (nitrite oxidation to nitrate) to generate energy [70]. Therefore, although the denitrification module was 100% complete and enriched at 28.5 °C, this likely reflects a lower potential for nitrite-derived energy production by Nitrospira, rather than denitrification, at 31.5 °C. With Thaumarchaeota as unique ammonia oxidisers, Nitrospira would have been deprived of an energy source, which explains the absence of these organisms in all but one sample under OW conditions.

The current study therefore illustrates the close association between these two microorganisms, which has been reported in several sponge hosts and recently reviewed [71]. Importantly, it further implies that the 3 °C increase in seawater temperature induced an imbalance in the nitrogen cycle, likely leading to an accumulation of toxic ammonia in the sponge tissue and potentially contributing to the decline in host health.

Elevated seawater temperature also negatively impacted the potential for the biosynthesis of amino acids (branch-chain and aromatic), co-factors and vitamins. Whilst amino acids act as building blocks of proteins, their derivatives can also carry other vital functions. For instance, glycine betaine acts as an osmoprotectant, which organisms such as sponges can also use for nitrogen storage [72], and it has been hypothesised that its production is needed to maintain a healthy symbiosis within coral holobionts [73]. Similarly, the compound folate is involved in processes ranging from the biosynthesis of DNA to the activation of cobalamin [74]. The conversion of folate into its derivatives allows the transport of one-carbon compounds in the cell as well as the conversion of serine into glycine. Sponge cells can obtain amino acids, cofactors and vitamins via feeding [71], but microbially derived compounds can provide a steady supply to the host [10]. Therefore, the reduced microbial potential for the biosynthesis of compounds as diverse as valine, tryptophane, glycine betaine or cobalamin likely deepened the nutrient and metabolic imbalance, affecting multiple aspects of cell functioning, and further weakening the holobiont.

### Defense against thermally-induced oxidative stress

The pentose phosphate metabolism plays a central role in cellular functioning and homoeostasis, and genes encoding these pathways were particularly enriched in sponges at 31.5 °C. The PPP is composed of two sections: (i) the oxPPP ensures the unidirectional breakdown of glucose-6-P into ribulose-5-P, generating two molecules of NADPH, which can be used for reductive biosynthesis or as an antioxidant, (ii) the non-oxidative branch of the PPP converts ribulose-5-P into ribose-5-phosphate (R5P), which forms the backbone of nucleic acids, but also various sugars such as erythrose-4-P, a precursor of vitamin B6 and aromatic amino acids [75, 76]. In parallel, the EDP allows organisms to by-pass the non-oxPPP by returning intermediates of this pathway to glycolysis, while generating reducing power in the form of NADPH [77, 78]. This compound is an antioxidant used to protect proteins from reactive oxygen species (ROS), and to repair DNA [76, 79, 80].

With respiration rates increasing at high temperatures in *S. flabelliformis* [29], it is likely that sponges were experiencing oxidative stress. In this context, while regulation of NADPH concentrations in response to increased ROS typically occurs at the transcriptomic level [76], it is possible that at high temperature, microbes possessing oxPPP and ED had a selective advantage compared to others, via a greater potential for defence against oxidative stress.

## Conclusion

**Fig. 5:**
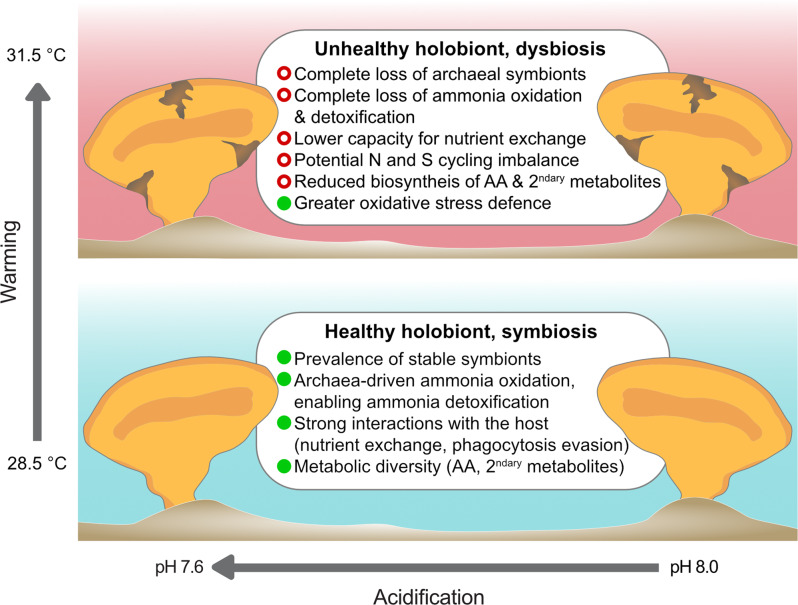
Overall impact of ocean warming and acidification on the *S. flabelliformis* holobiont. Green dots and red circles represent positive and negative features for the symbiosis, respectively. Ocean acidification had no impact, whilst ocean warming impacted sponge health and microbial functions, regardless of pH.

Here we showed that, while tolerant to OA, the widespread and ecologically important reef sponge *S. flabelliformis* is highly sensitive to OW, with microbial dysbiosis and host necrosis occurring at 31.5 °C. These seawater temperatures correspond to marine heatwave conditions already recorded in Australia and predicted to occur more frequently in the coming decades [14]. Under OW, the relationship between the host and its microbiome breaks down at both the community and molecular levels in visibly healthy tissue (Fig. 5). The dramatic shift in community composition, including the loss of Thaumarchaeota, reduces the presence of highly diverse metabolisms, in particular through a loss of capacity for ammonia detoxification and reduced potential for biosynthesis of amino acids, co-factors and vitamins. Regardless of an apparent selective advantage for microbes capable of producing antioxidants at elevated temperature, the symbiosis becomes substantially compromised under OW, with adverse outcomes for holobiont health.

Considering the importance of sponges in marine ecosystems and the crucial role of microorganisms for these animals, further research exploring the thermal tolerance of sponge holobionts is urgently needed, in particular to better understand: (i) thresholds for symbiosis breakdown, (ii) the capacity for holobionts to recover from dysbiosis and (iii) the extent and timeline of “recolonisation” by symbionts. We anticipate that, similar to the field of coral bleaching and symbiosis research [81–83], multi-disciplinary approaches combining holobiont data and spanning physiology, microscopy, gene expression, metabolomics and investigation of nutrient exchange with techniques such as Nano-SIMS, will be required to provide a mechanistic understanding of sponge dysbiosis in a warming ocean.

## Supplementary information

Supplementary material

## Data Availability

Raw sequence data are available from the NCBI SRA under project PRJNA588103. The assemblies are available from IMG/MER (https://img.jgi.doe.gov/cgi-bin/m/main.cgi), see Table S3 for IDs.
