# Supplementary material for: Future ocean conditions induce necrosis, microbial dysbiosis and nutrient cycling imbalance in the reef sponge Stylissa flabelliformis
Source: ISME Commun. 2023 Jun 14;3:53. doi: 10.1038/s43705-023-00247-3 (PMC10264452; doi:10.1038/s43705-023-00247-3)
Supplement: Supplementary file 1 — Supplementary material [file 43705_2023_247_MOESM1_ESM.pdf]

Future ocean conditions induce necrosis, microbial dysbiosis and nutrient cycling imbalance in the reef sponge *Stylissa flabelliformis*.

Supplementary material

Emmanuelle S. Botté, Holly Bennett, J. Pamela Engelberts, Torsten Thomas, James J. Bell, Nicole S. Webster, Heidi M. Luter

Table S1. Temperature and pH during experimental ramping. Values are ml/min of CO_2 for each pH treatment. Adapted from Bennett et al., 2017.

Day	Time	Temperature		28.5 °C		31.5 °C	
		28.5 °C	31.5 °C	pH 8.0	pH 7.6	pH 8.0	pH 7.6
1	17:00	27.3 °C	27.3 °C				
	20:00	27.6 °C	27.6 °C	--	3	--	2.8
	23:00	27.9 °C	29.9 °C				
2	8:00	28.1 °C	28.1 °C				
	11:00	28.3 °C	28.3 °C				
	14:00	28.5 °C	28.5 °C				
	17:00		28.8 °C	--	6.1	--	5.6
	20:00		29.1 °C				
	23:00		29.3 °C				
3	8:00		29.6 °C				
	11:00		29.9 °C	--	9.2	--	8.2
	14:00		30.2 °C				
	17:00		30.5 °C				
	20:00		30.8 °C				
	23:00		31.1 °C				
4	8:00		31.3 °C				
	11:00		31.5 °C				

Table S2. Measurements of temperature and pH. Data are derived from weekly in-tank measurements (n = 22 sampling periods) and show average (SD). Adapted from Bennett et al., 2017.

Treatment	Tank replicate	Temperature	pH	Number of samples taken
28.5°C/ pH 8.0	1	28.67 (0.10)	8.01 (0.05)	n = 0
28.5°C/ pH 8.0	2	28.60 (0.20)	8.00 (0.05)	n = 1
28.5°C/ pH 8.0	3	28.61 (0.16)	8.01 (0.05)	n = 2
28.5°C/ pH 7.6	1	28.49 (0.13)	7.66 (0.05)	n = 1
28.5°C/ pH 7.6	2	28.48 (0.18)	7.69 (0.08)	n = 2
28.5°C/ pH 7.6	3	28.50 (0.22)	7.69 (0.08)	n = 0
31.5°C/ pH 8.0	1	31.45 (0.18)	8.01 (0.11)	n = 2
31.5°C/ pH 8.0	2	31.64 (0.17)	8.03 (0.05)	n = 1
31.5°C/ pH 8.0	3	31.45 (0.15)	8.02 (0.06)	n = 0
31.5°C/ pH 7.6	1	31.41 (0.19)	7.66 (0.05)	n = 1
31.5°C/ pH 7.6	2	31.58 (0.15)	7.66 (0.08)	n = 1
31.5°C/ pH 7.6	3	31.45 (0.20)	7.64 (0.07)	n = 1

Table S3. Statistics throughout reads processing.

Sample	Treatment	IMG ID	Number of raw reads	% of reads kept after quality trimming	Number of contigs assembled (mean length)	Number of contigs after filtering	Mean contig length
S01	28.5 °C pH 8.0	3300022766	2513400	99.8	94216	81226 (86.2%)	609 bp
S04	28.5 °C pH 8.0	3300022793	2041680	99.6	71013	63074 (88.8%)	603 bp
S10	28.5 °C pH 8.0	3300022784	2153738	99.7	64937	56039 (86.3%)	658 bp
S02	28.5 °C pH 7.6	3300022794	2234386	99.8	104980	94199 (89.7%)	599 bp
S03	28.5 °C pH 7.6	3300022797	2274734	99.8	93746	82812 (88.3%)	609 bp
S12	28.5 °C pH 7.6	3300022768	2406692	99.8	89667	74138 (82.7%)	558 bp
S06	31.5 °C pH 8.0	3300023277	2240266	99.7	105402	98964 (93.9%)	524 bp
S07	31.5 °C pH 8.0	3300022814	2422394	99.6	122973	113791 (92.5%)	556 bp
S11	31.5 °C pH 8.0	3300022765	2282472	99.7	128491	119094 (92.7%)	544 bp
S05	31.5 °C pH 7.6	3300022791	1979566	99.5	75667	70963 (93.8%)	553 bp
S08	31.5 °C pH 7.6	3300022798	2205930	99.8	91308	86082 (94.3%)	631 bp
S09	31.5 °C pH 7.6	3300022763	2368540	99.6	74611	65261 (87.5%)	639 bp

Table S4. Taxa with significantly different relative abundances between 28.5 °C and 31.5 °C as identified using AncomBC. Results shown for taxa with relative abundance > 1%. Thaumarch: Thaumarchaeota

Taxon	Relative abundance at 28.5 °C (%)	Relative abundance at 31.5 °C (%)
Archaea; p_Crenarchaeota; c_Thaumarch.; o_Cenarchaeales; f_Cenarchaeaceae	1.35	0.00
Archaea; p_Crenarchaeota; c_Thaumarch.; o_Cenarchaeales; f_Cenarchaeaceae; g_Cenarchaeum; s_symbiosum	11.00	0.00
Archaea; p_Crenarchaeota; c_Thaumarch.; o_Cenarchaeales; f_Cenarchaeaceae; g_Nitrosopumilus	2.67	0.00
Bacteria	3.99	10.61
Bacteria; p_Chlamydiae; c_Chlamydiia	0.33	1.58
Bacteria; p_Chlamydiae; c_Chlamydiia; o_Chlamydiales	1.07	4.92
Bacteria; p_Chloroflexi	0.05	1.05
Bacteria; p_Chloroflexi; c_Anaerolineae; o_Anaerolineales; f_Anaerolinaceae; g_T78	0.85	3.52
Bacteria; p_Nitrospirae; c_Nitrospira; o_Nitrospirales; f_Nitrospiraceae	1.51	0
Bacteria; p_OD1; c_ZB2	0.59	1.79
Bacteria; p_Proteobacteria; c_Alphaproteobacteria	0.12	1.07
Bacteria; p_Proteobacteria; c_Alphaproteobacteria; o_Rhodobacterales; f_Rhodobacteraceae	0.14	3.28
Bacteria; p_Proteobacteria; c_Alphaproteobacteria; o_Rickettsiales	7.82	21.35
Bacteria; p_Proteobacteria; c_Gammaproteobacteria	5.68	1.25
Bacteria; p_Proteobacteria; c_Gammaproteobacteria; o_[Marinicellales]; f_[Marinicellaceae]	2.91	0.20
Bacteria; p_Proteobacteria; c_Gammaproteobacteria; o_Thiohalorhabdales	21.27	2.18

Table S5. Distribution of mod_{70} with significantly different relative abundances between 28.5 °C and 31.5 °C as identified using edgeR.

Modules categories	Number of modules	Proportion of the total (%)
Energy metabolism	11	50.6
Carbohydrate metabolism	9	
Amino acid metabolism	8	
Metabolism of cofactors and vitamins	5	
Lipid metabolism	5	
Nucleotide metabolism	2	
Transport systems	23	39.2
Other environmental information and processing	5	
Genetic information processing	8	
Others	4	
Total	***79***	

Table S6. IMG taxonomy assignment for module related to rhamnose biosynthesis.

Module - Pathway	KO/Gene/ Function	Dominant taxa at 28.5 °C (%)	Dominant taxa at 31.5 °C (%)
M00793 – Rhamnose biosynthesis	K00067	Alphaproteobacteria (76.85)	Alphaproteobacteria (63.39)
	K00973	Alphaproteobacteria (57.73)	Alphaproteobacteria (25.24)
	K01710	No dominant	No dominant
	K01790	Alphaproteobacteria (66.67)	Alphaproteobacteria (33)

Table S7. IMG taxonomy assignment from IMG for KOs related to metabolism. The most abundant (or important) taxon is shown first. Italics represent taxa that are not dominant but important for the results' interpretation. "Absent" means that this KO was not found at that particular treatment.

Module - Pathway	KO term & Name in KEGG	Dominant taxa at 28.5 °C (%)	Dominant taxa at 31.5 °C (%)
**M00159 (Vtype ATP-ase)**	K02107 V/A-type H+/Na+-transporting ATPase subunit G/H	Thaumarch.	Absent
	K02117 V/A-type H+/Na+-transporting ATPase subunit A (EC 7.1.2.2 7.2.2.1)	Thaumarch.	2 reads in 1 sample
	K02118 V/A-type H+/Na+-transporting ATPase subunit B	Thaumarch.	Absent
	K02119 V/A-type H+/Na+-transporting ATPase subunit C	Thaumarch.	Absent
	K02120 V/A-type H+/Na+-transporting ATPase subunit D	Thaumarch.	Absent
	K02121	Thaumarch.	Absent

	V/A-type H+/Na+-transporting ATPase subunit E		
	K02122<br>V/A-type H+/Na+-transporting ATPase subunit F	Thaumarch.	Absent
	K02123<br>V/A-type H+/Na+-transporting ATPase subunit I	Thaumarch.	3 reads in 1 sample
	K02124<br>V/A-type H+/Na+-transporting ATPase subunit K	Thaumarch.	3 reads in 1 sample
**M00149 (Succinate dehydrogenase)**	K00239<br>Succinate dehydrogenase / fumarate reductase, flavoprotein subunit (EC 1.3.5.1 ; 1.3.5.4)	No dominant	No dominant
	K00240<br>Succinate dehydrogenase / fumarate reductase, iron-sulfur subunit (EC 1.3.5.1; 1.3.5.4)	Gammaprot. (42.8)<br>Thaumarch. (22.8)<br>*Alphaprot. (0.63)*	Gammaprot. (44.3)<br>Alphaprot. (37.4)<br>*Thaumarch. (0)*
	K00241<br>Succinate dehydrogenase / fumarate reductase, cytochrome b subunit	Thaumarch. (27.9)<br>Gammaprot. (16.4)	Gammaprot. (48.6)<br>Alphaprot. (33.9)<br>*Thaumarch. (0)*
	K00242<br>Succinate dehydrogenase / fumarate reductase, membrane anchor subunit	Thaumarch. (28.2)<br>Gammaprot. (18.5)	Gammaprot. (49.1)<br>*Thaumarch. (0)*
**M00528 (Nitrification – ammonia oxidation)**	K10944<br>Ammonia monooxygenase subunit A (EC 1.14.99.39)	Thaumarch.	Absent
	K10945<br>Ammonia monooxygenase subunit B	Thaumarch.	Absent
	K10946<br>Ammonia monooxygenase subunit C	Thaumarch.	Absent
**M00529 ("Denitrification" - see Discussion section)**	K00368<br>Nitrite reductase (NO-forming) (EC:1.7.2.1)	Gammaprot. (32.1)<br>Thaumarch. (28.6)	No dominant<br>*Thaumarch. (0)*
	K00370<br>Nitrate reductase / nitrite oxidoreductase, alpha subunit (EC:1.7.5.1 1.7.99.-)	Alphaprot. (56.7)<br>Nitrospira (3.6)	Alphaprot. (49.7)<br>*Nitrospira (0)*
	K00371<br>Nitrate reductase / nitrite oxidoreductase, beta subunit (EC:1.7.5.1 1.7.99.-)	Alphaprot. (94.8)<br>Nitrospira (4.4)	Alphaprot. (100)<br>*Nitrospira (0)*
	K00374<br>Nitrate reductase gamma subunit (EC:1.7.5.1 1.7.99.-)	Alphaprot. (64)	Alphaprot. (84)
	K00376	No dominant	No dominant

	Nitrous-oxide reductase (EC:1.7.2.4)		
**M00596 (Dissimilatory sulfate reduction)**	K00394 Adenylylsulfate reductase, subunit A (EC:1.8.99.2)	Gammaprot. (42.8)<br>Betaprot. (21)	Gammaprot. (35)<br>*Betaprot. (0)*
	K00395 Adenylylsulfate reductase, subunit B (EC:1.8.99.2)	Gammaprot. (31.6)<br>Betaprot. (8.1)	Gammaprot. (10)<br>Betaprot. (4)
	K11180 Dissimilatory sulfite reductase alpha subunit (EC:1.8.99.5)	Gammaprot. (93.1)	Gammaprot. (33.8)
	K11181 Dissimilatory sulfite reductase beta subunit (EC:1.8.99.5)	Gammaprot. (80.1)	Gammaprot. (92.2)
**M00006 (PPP)**	K15916 Glucose/mannose-6-phosphate isomerase (EC:5.3.1.9 5.3.1.8)	Thaumarch. (52.8)	Absent
	K01808 Ribose 5-phosphate isomerase B (EC:5.3.1.6)	Absent	Alphaprot. (75)
	K000336-phosphogluconate dehydrogenase (EC:1.1.1.44 1.1.1.343)	Nitrospira (94.1)	*Nitrospira (0)*
	K00036 Glucose-6-phosphate 1-dehydrogenase (EC:1.1.1.49 1.1.1.363)	Nitrospira (19)<br>Alphaprot. (15.4)<br>Gammaprot. (2.7)	Alphaprot. (16.9)<br>Gammaprot. (7)<br>*Nitrospira (0)*
**M00019 (Valine biosynthesis)**	K01652 Acetolactate synthase I/II/III large subunit (EC:2.2.1.6)	Rhodobact. (20.7)<br>Chromatiales (13)<br>Thaumarch. (3)<br>Nitrospira (0.3)	Rhodobact. (19.9)<br>Chromatiales (5.3)<br>*Thaumarch. (0)*<br>*Nitrospira (0)*
	K01653 Acetolactate synthase I/III small subunit (EC:2.2.1.6)	Gammaprot. (52)<br>Thaumarch. (15.8)	Gammaprot. (33.6)<br>*Thaumarch. (0)*
	K00053 Ketol-acid reductoisomerase (EC:1.1.1.86)	Chromatiales (22.7)<br>Archaea (10.4)	Chromatiales (6.1)<br>*Archaea (0)*
	K01687 Dihydroxy-acid dehydratase (EC:4.2.1.9)	Gammprot. (14.9)<br>Thaumarch. (4.2)<br>Rhodobact. (3.6)	Gammprot. (18.7)<br>Rhodbacterales (9)<br>*Thaumarch. (0)*
	K00826 Branched-chain amino acid aminotransferase (EC:2.6.1.42)	Gammprot. (29.1)<br>Rhodobact. (14.6)<br>Thaumarch. (3)	Gammprot. (27)<br>Rhodobact. (12.1)<br>*Thaumarch. (0)*
**M00023 (tryptophan biosynthesis)**	K01696 Tryptophan synthase beta chain (EC:4.2.1.20)	Alphaprot. (37.8)<br>Thaumarch. (26.7)<br>Gammaprot. (2.2)	Gammaprot. (48)<br>Alphaprot. (11)<br>*Thaumarch. (0)*
	K01609 Indole-3-glycerol phosphate synthase (EC:4.1.1.48)	Thaumarch. (38.4)<br>Gammaprot. (1.9)	Alphaprot. (46)<br>Betaprot. (7.7)<br>*Thaumarch. (0)*
	K00766 Anthranilate phosphoribosyltransferase (EC:2.4.2.18)	Gammaprot. (35.5)<br>Thaumarch. (15.9)<br>Nitrospira (5.8)<br>*Alphaprot. (0)*	Gammaprot. (25.8)<br>Alphaprot. (6.7)<br>*Thaumarch. (0)*<br>*Nitrospira (0)*

	K01658<br>Anthranilate synthase component II (EC:4.1.3.27)	Betaprot. (16)<br>Gammaprot. (14)<br>Archaea (12)	Gammaprot. (40)<br>*Archaea (0)*
**M00127 (thiamine biosynthesis)**	K03151<br>tRNA uracil 4-sulfurtransferase (EC:2.8.1.4)	Bacteroidetes (13.5)<br>Nitrospira (13.5)	Alphaprot. (7.2)<br>Bacteroidetes (3.6)
	K03154<br>Sulfur carrier protein		
	K14153<br>Hydroxymethylpyrimidine kinase / phosphomethylpyrimidine kinase / thiamine-phosphate diphosphorylase (EC:2.7.1.49; 2.7.4.7 2.5.1.3)	Gammaprot. (33.7) Nitrospira (13.6)	Gammaprot. (42)<br>Alphaprot. (4.2)
	K00788<br>Thiamine-phosphate pyrophosphorylase (EC:2.5.1.3)	Nitrospira (16.7)<br>Gammaprot. (11.9)	Alphaprot. (9.3)<br>Gammaprot. (4.6)
	K03147<br>Phosphomethylpyrimidine synthase (EC:4.1.99.17)	Thaumarch. (43)<br>Nitrospira (7.9)<br>Alphaprot. (1.3)	Alphaprot. (76.2)<br>Gammaprot. (76)
	K03149<br>Thiazole synthase (EC:2.8.1.10)	Gammaprot. (44.7)	Gammaprot. (38.6)<br>Alphaprot. (9.4)
**M00555 (Glycine betaine biosynthesis)**	K00108<br>Choline dehydrogenase (EC:1.1.99.1)	Gammaprot. (40)<br>Alphaprot. (12.2)	Alphaprot. (36.5)<br>Gammaprot. (7.5)
	K00499 choline monooxygenase (EC 1.14.15.7)	Gammaprot. (46.2)<br>Alphaprot. (9.8)	Alphaprot. (47.9)<br>Gammaprot. (11.6)
**M00122(Cobalamin biosynthesis)**	K00798<br>Cob(I)alamin adenosyltransferase (EC 2.5.1.17)	Thaumarch. (9.5)	*Thaumarch. (0)*
	K02227<br>Adenosylcobinamide-phosphate synthase (EC 6.3.1.10)	Thaumarch. (20)	*Thaumarch. (0)*
	K00232<br>Adenosylcobyric acid synthase (EC 6.3.5.10)	Thaumarch. (9.5)	*Thaumarch. (0)*
	K00233 Adenosylcobinamide-GDP ribazoletransferase (EC:2.7.8.26)	Thaumarch. (16)	Thaumarch. (1)
**M00117 (Ubiquinone biosynthesis)**	K03182<br>4-hydroxy-3-polyprenylbenzoate decarboxylase (EC:4.1.1.98)	Thaumarch. (57.9)	*Thaumarch. (0)*
	K03183<br>Demethylmenaquinone methyltransferase / 2-methoxy-6-polyprenyl-1,4-benzoquinol methylase (EC:2.1.1.163; 2.1.1.201)	Thaumarch. (17)	*Thaumarch. (0)*
	K03186<br>Flavin prenyltransferase (EC:2.5.1.129)	Thaumarch. (13.9)	*Thaumarch. (0)*

**M00140 (C1 unit-folate)**	K00600 Glycine hydroxymethyltransferase (EC:2.1.2.1)	Thaumarch. (12.8)	*Thaumarch. (0)*
	K01491 Methylenetetrahydrofolate dehydrogenase (NADP+) / methenyltetrahydrofolate cyclohydrolase (EC 1.5.1.5; 3.5.4.9)	Thaumarch. (16.4)	*Thaumarch. (0)*

Supplementary notes

Thaumarchaeota taxonomy

The Thaumarchaeota present at 28.5 °C were primarily identified as members of the family Cenarchaeaceae or species *Cenarchaeum* or *Nitrosopumilus* (Supplementary data spreadsheet "SOM_taxonomy"). These represent the overwhelming majority of Thaumarchaeota found in other sponge species: 99.7% of the unique Thaumarchaeota OTUs recovered from 81 marine sponge species were assigned to Cenarchaeaceae at the family level, and 91% of these OTUs were assigned to either *Cenarchaeum* or *Nitrosopumilus* at the species level (Thomas et al., 2016, Supplementary Data).

Assignment of KOs into higher level categories (Fig 2c)

The 913 KOs that were significantly different between 28.5 and 31.5 °C are represented on Fig. 2a and Fig 2b. KOs were subsequently assigned to higher level categories (Brite hierarchy level B) for meaningful data interpretation. KOs often represent genes that belong to several pathways, and they can therefore fall into several categories. Consequently, total counts for the number of KOs, after assigning KOs to categories, amounted to 1,805, meaning that each KO fell in 2 categories on average. Meanwhile, the number of unique KOs assigned to higher level categories was 910, meaning that 3 KOs were not assigned to any category; they were therefore not represented in Fig 2.

Out of the 1,805 counts for assignments to higher level categories, 171 (or 9.5%) contained KOs assigned to "Poorly characterized" or "Unidentified" pathways, 152 (or 8.4%) to pathways pertaining to "Human diseases" or "Organismal systems", and manual curation identified 36 additional counts (2%) which assigned KOs to eukaryotic processes within KEGG pathways (eg. "Peroxisome" or "Ribosomal genesis in eukaryotes"); all were excluded from Fig. 2.

The remaining 80.1% of counts represented KOs assigned to “Brite hierarchies” (489 counts or 27.1%) and to KEGG pathways (957 counts or 53%). Brite Hierarchies sub-categories were “Protein families: metabolism”, “Protein families: genetic information and processes” and “Protein families: signalling and cellular processes”. Since these categories overlap with KEGG categories traditionally used in environmental metagenomic studies, only KEGG pathways were represented on Fig 2.

Carbohydrate metabolism

Enrichment was found with module M00307 representing pyruvate oxidation (**Fig. 4**). This module encompasses the reaction catalysed by *i)* the pyruvate dehydrogenase complex during aerobic respiration (ECs 1.2.4.1, 1.8.1.4 and 2.3.1.12), which produces acetyl-CoA, NADH and CO_2, and by *ii)* pyruvate:ferredoxin oxidoreductases (PFOR, EC 1.2.7.1), which only generate acetyl-CoA. Whilst the functional potential for pyruvate oxidation was overall relatively mildly enhanced at 31.5 °C (with a 1.6-times increase), four of the five genes encoding the pyruvate dehydrogenase complex were 1.4- to 7.7-times more abundant at 31.5 °C, whereas three of the four genes encoding PFORs were found exclusively under control conditions and were carried by Nitrospira, which again highlights the strong impact of the taxonomic shift on the functional potential of the microbiomes examined.
